# Opinions, Attitudes and Factors Related to SARS-CoV-2 Vaccine Uptake in Eight South American Countries

**DOI:** 10.3390/vaccines11111660

**Published:** 2023-10-30

**Authors:** Analía Urueña, Ricardo Machado, Juarez Cunha, Clara López Colmano, Carolina Rancaño, Renato Kfouri, Catalina Pírez, Pablo Bonvehí, Mario Calvo, Robinson Cuadros, Greta Muñoz, Mónica Rodríguez, Jaime Torres, Florencia Cahn, Isabella Ballalai

**Affiliations:** 1 Centro de Estudios para la Prevención y Control de Enfermedades Transmisibles, Universidad Isalud, Venezuela 931, Ciudad Autónoma de Buenos Aires C1095AAS, Argentina; 2 Sociedad Argentina de Vacunología y Epidemiología (SAVE), Ciudad Autónoma de Buenos Aires, Argentina; 3 Sociedade Brasileira de Imunizações (Sbim), R. Luís Coelho, 308-Consolação, São Paulo 01309-000, SP, Brazilisabellaballalai@gmail.com (I.B.); 4 Sociedad Uruguaya de Pediatría, Comité de Infectología y Vacunas, Lord Ponsonby 2410, Montevideo 11600, Uruguay; 5 Servicio de Infectología, Hospital Universitario CEMIC, Dr. Ricardo Balbín 4459, Ciudad Autónoma de Buenos Aires C1430ABC, Argentina; 6 Instituto de Medicina, Facultad de Medicina, Universidad Austral de Chile, Coronel Santiago Bueras 1003, Valdivia 5110566, Chile; macalvo@uach.cl; 7 Asociación Internacional de Gerontología y Geriatría, Comité Latinoamericano y del Caribe, Carrera 7C Bis 139-17, Bogotá 110121, Colombia; rcuadros@cafam.com.co; 8 Sociedad Ecuatoriana de Pediatría (SEP), Av. Naciones Unidas E2-17 e, Quito 170135, Ecuador; 9 Hospital Central, Instituto de Previsión Social, PCH9+4RX, Santísimo Sacramento, Asunción 1519, Paraguay; 10 Sección de Enfermedades Infecciosas, Instituto de Medicina Tropical, Universidad Central de Venezuela, Caracas 1040, Venezuela

**Keywords:** vaccine, COVID-19, coronavirus, vaccine hesitancy

## Abstract

This article presents attitudes and practices regarding COVID-19 vaccination in the South American population. The study collected data from a self-administered survey distributed through social media platforms between February and April 2022 (*N* = 6555). The survey included questions related to participants’ sociodemographic background, flu vaccination practices, sources of information about COVID-19, and opinions regarding pandemic management and vaccination against SARS-CoV-2. The respondents agreed with the statement that COVID-19 vaccines were necessary (86.4%), effective (79.8%), safe (79.1%), and should be mandatory (64%). Overall, 83.4% accepted vaccination and 12.3% refused it completely. Main rejection reasons were safety (65.8%) and efficacy (54.9%) issues, and rushed development and approvals (49.1%). Vaccine uptake was associated with being ≥60 years, being a healthcare worker, previous influenza vaccine uptake, adherence to preventive measures, the death of ≥1 close people from COVID-19, and being informed through mass media or health authorities’ channels. Vaccine uptake inversely correlated with male gender, low educational level, and use of closed social networks for COVID-19 information purposes. This study provides valuable insights into COVID-19 vaccination attitudes and practices in South America that may be used to promote vaccine uptake in the region. Higher COVID-19 vaccination acceptance among people with previously acquired prevention habits reinforces the importance of routine health promotion strategies.

## 1. Introduction

Throughout human history, infectious diseases such as smallpox, polio, measles, rubella, diphtheria, and many others have resulted in millions of deaths worldwide [1]. The global spread of these diseases has led to significant mortality, emphasizing the need to implement effective strategies to combat them. In the last century, the control of these diseases has been mainly achieved through immunization [2]. In 1796, for the first time, Edward Jenner inoculated an eight-year-old boy with bovine smallpox pustules. Later, when he injected him with the human smallpox virus, the boy was neither infected nor had symptoms. Therefore, Jenner’s work represented the first attempt to control an infectious disease through the deliberate use of vaccination [3]. Since then, immunization by vaccines has had a significant impact on public health globally by reducing morbidity and mortality caused by life-threatening infectious diseases [4]. The impact of vaccines is broad and far-reaching, not only in terms of health but also in generating multiple economic and social benefits [5].

According to the World Health Organization (WHO), vaccine hesitancy is the reluctance or refusal to vaccinate despite vaccine availability [6]. This phenomenon, which contributes to suboptimal vaccination coverage and increases the risk of a resurgence of preventable diseases, has been identified as one of the top ten threats to global health by the World Health Organization (WHO), as it implies a high risk to reverse the progress made in the fight against vaccine-preventable diseases [6]. Recently, while the world was facing one of the worst health catastrophes in history caused by COVID-19, the infodemic (the rapid spread and amplification of vast amounts of invalid information on the internet) [7] and misinformation generated as much fear as the virus itself, leading many people to reject vaccination—the most effective strategy to control the disease. Thus, knowing that this pandemic may not be the last one, it is necessary to reinforce vaccine confidence.

New vaccines tend to cause more indecision than older vaccines. Although public anxiety and concern about vaccine safety have always existed, the rise of social media has allowed for the spread of misinformation to a much wider audience, affecting vaccination attitudes, opinions, and uptake [8]. Moreover, the resurgence of anti-vaccine groups and conspiracy theories and the difficulties in the production, purchase, and management of vaccines by governments have produced new myths and beliefs that increase fear of vaccination [9]. In this regard, in a large survey from 2021 that included 23,000 people from 23 countries, country vaccination rates were negatively associated with vaccine hesitancy [10]. It has been suggested that the COVID-19 pandemic highlighted the relevance of vaccines to many people who trust vaccination but may have negatively affected those who were hesitant or misinformed [11].

The COVID-19 pandemic has also exposed the inequality of access to vaccines in several low- and middle-income countries. The WHO stated in May 2022 that nearly 18 months after the first administration of a COVID-19 vaccine, only 16% of people in low-income countries had received a single vaccine dose, compared to 80% in high-income countries. The low vaccination coverage in these countries is attributed to inadequate provision and distribution of vaccines [12]. In this case, it is important to mention that the social, economic, and political context limits access to vaccines and deepens inequality even further [13].

Regarding the case of Latin America and the Caribbean, an evidence-based study that assessed barriers to regular vaccinations in the region found that individual/group perceptions of the vaccine and the social environment are the main factors that prevent optimal vaccination. In these countries, there are other barriers to vaccination acceptance, including unfavorable socioeconomic factors, low level of education, lack of knowledge about the diseases and their vaccines, and religious and cultural beliefs [14]. Furthermore, in countries such as Colombia, Ecuador, and Venezuela, a variety of barriers to COVID-19 vaccination have been identified, ranging from structural issues to the belief in myths and misconceptions. [15]. Interestingly, an experiment conducted in six Latin American countries observed that vaccine hesitancy can be reduced by providing basic information about vaccines to the population, encouraging individuals to believe they can be part of a successful collective effort, and leveraging the reputation benefits they expect to receive by getting vaccinated [16]. These findings underscore the importance of identifying the causes of vaccine hesitancy in certain sectors of the population. Thus, by determining these barriers, specific and multifaceted communication strategies can be designed to address this issue and assist countries in combating vaccine hesitancy. The COVID-19 infection has not only impacted Latin America, causing thousands of deaths, but has also triggered an emergency derived from the combination of viral infection and non-communicable diseases rooted in the social and economic inequalities of this region [17]. Currently, although vaccination is available and demonstrated to be the most effective tool to prevent severe forms of COVID-19 disease and deaths, adherence is still incomplete, and misinformation and doubts persist. As new SARS-CoV-2 variants continue to emerge, and personal protection measures are relaxed, the impact of future possible variants remains unpredictable, highlighting the importance of increasing vaccine equity and confidence [18]. In this sense, the development of public health strategies that counteract the rejection of vaccination will depend on the identification of multiple factors that vary according to the social, political, economic, and cultural context of each country or region. For this reason, in this study, we attempt to characterize the attitudes and practices related to COVID-19 vaccination among the general population and health care workers in eight South American countries that may be of use for targeted interventions to promote vaccine confidence and uptake in the region.

## 2. Materials and Methods

### 2.1. Ethical Considerations and Informed Consent

This research was conducted by Confianza en las vacunas Latinoamérica, an international group of professionals that works in vaccine confidence in Latin America. The aim of this group is to generate a positive impact on health professionals and the population in general, through key messages and communication strategies based on local evidence.

This study was approved by the Institutional Review Board (IRB) of Fundación Huésped through the Computerized Registry Platform for Health Research in Buenos Aires (PRIISA): Registry N° 6204. The survey was administered by MAAS Marketing Asesorado. Participants were informed about the objectives of this study and were asked to explicitly state their desire to participate in it.

### 2.2. Study Design and Participants

A descriptive cross-sectional study was designed and conducted. A general population of both sexes, and particularly health care workers (HCWs), were recruited as participants. Inclusion criteria for the study were: individuals over 15 years old from the following South American countries: Argentina, Brazil, Chile, Colombia, Ecuador, Paraguay, Uruguay, and Venezuela. The target sample to be recruited were 7200 valid respondents (900 per country) allocated according to the United Nations estimates’ population distribution in terms of region, gender, and age [19]. Age strata were stratified as 15–19, 20–29, 30–39, 40–49, 50–59, and 60 years and older. Regions with less than 3% of the population were excluded in order to obtain the same consistency between countries and to better leverage advertising performance.

We created sponsored outreach campaigns on social media (Facebook and Instagram), segmented by age group, gender, and region/state for each of the eight countries. For each country, there was a specific advertisement with the following text: “Do you live in [customized country]? We want to know what you think about COVID-19 vaccines! Answer our survey in 5 min. COVID-19 Vaccines”. As we completed each audience quote required for the sample, those audiences were removed, and we kept only the ads for the remaining audiences until all quotes were completed.

The self-administered web-based survey included closed-ended questions about attitudes and practices regarding COVID-19 vaccination (see Supplementary Materials). The questionnaire was developed with the GANDIA software that allows for its design and the analysis of the collected data. 

To encourage the respondents to provide more truthful and honest answers, an online format was chosen, which allowed anonymity and the possibility of conducting fast and large-scale data collection. 

Questionnaire data collection took place between February and April 2022. At that time, most countries in South America were passing through the third COVID-19 wave, with a high predominance of the Omicron variant. The proportion of people vaccinated with at least one dose of the COVID-19 vaccine in the analyzed countries was, at that time, close to 80% or above in most countries, except for Paraguay, where they just exceeded 50% [20]. The instrument was designed by a group of experts from Confianza en las Vacunas Latinoamérica and consisted of 29 questions that assessed: (1) Participants’ sociodemographic background (age, gender, educational level, country of origin, employment situation, healthcare worker occupation, self-history of COVID-19 infection, and close person death from COVID-19); (2) Sources of information used to learn about the COVID-19 pandemic and vaccination against SARS-CoV-2; (3) Previous practices towards flu vaccination; (4) Opinions regarding the pandemic management in their countries, including health authorities’ recommendations; (5) Perceptions regarding necessity, safety, efficacy and compulsory SARS-CoV-2 vaccination; (6) Their own SARS-CoV-2 vaccination status, including the type of vaccine received; (7) The reasons that led to vaccination acceptance or rejection; and (8) Acceptance or rejection to a particular vaccine type. Responses to many statements were answered on Likert scales ranging from “strongly agree” to “completely disagree”. No identifiable personal information was asked or stored. The questionnaire, translated into English, is downloadable online as a supplementary file. 

The final sample included 6555 valid questionnaires. The sampling error for the total sample was 1.19%, calculated based on a *p* = 50% and a confidence coefficient of 95%. The results were weighted to express the real population proportionality of each country within the region and of each age segment within each country.

### 2.3. Statistical Analysis 

All numerical data were expressed as mean ± standard deviation (SD). Categorical variables were described using percentages and analyzed using the χ^2^-test with Yates correction. Odds ratios (ORs) and their 95% confidence intervals (CIs) were estimated.

*p*-values less than 0.05 were considered statistically significant. The data collected from the questionnaire were analyzed with GANDIA software version 4.42313.5. Statistical analysis was performed using GraphPad Prism statistics software version 9.0.0. 

## 3. Results

### 3.1. Participants’ Demographic Characteristics and COVID-19-Related Variables

The principal characteristics of the participants are summarized in Table 1. In this study, 6555 subjects were surveyed, of which 52% were females. The percentage of men and women was similar among the different countries and most of them resided in urban areas as the survey was tailored to districts where at least 3% of the total population of each country was concentrated. The age distribution of the participants was 15–19 years old (10%), 20–29 years old (20.7%), 30–39 years old (20%), 40–59 years old (31.2%), and over 60 years old (18%). More than half of the participants (65%) reached tertiary/university education. As the survey was conducted online, the studied sample comprised exclusively people with internet access. Surveyed individuals were mostly employees (46%). Moreover, 6% were HCWs, 17% were students, 20% were housewives/retired and 10.7% were unemployed people. Overall, 75% of the participants indicated that they received the flu vaccine at least once. From the total, 40.7% indicated they had COVID-19 (once or more). From these, most subjects referred only mild symptoms (86.4%), 9.6% recorded not having symptoms at all and 4% reported requiring hospitalization. Lastly, more than 40% of the overall respondents answered that they had suffered the loss of a close person due to death by COVID-19 (Table 1).

### 3.2. Sources of COVID-19 Information 

Most participants obtained their information from open social networks (52.8%), TV/radio newscasts (51.7%) and internet news portals (47.8%). The first two sources were the most used in all countries assessed except for Brazil, where the use of internet news portals prevailed over open social networks (51.8% vs. 48.2%, respectively). Official channels of public health authorities were the fourth most popular source of information, selected by 43.3% of the total sample, and reached third place in Argentina, Colombia, Ecuador, Paraguay and Uruguay. Closed social networks (WhatsApp, Telegram) were found to be additional sources for 16.1% of the participants, and this proportion was much higher in Chile and Venezuela (28.5% and 25.3%, respectively) (Table 1). 

In order to further explore the demographic characteristics or professional activities of the participants and the different media they adopt to obtain information about the topic, it was observed that adolescents and young adults most frequently chose open social networks (76%, 59% and 57% in the 15–19, 20–29, and 30–39 years old groups, respectively), in contrast to older adults, for whom these media represented less than a 45% and the most frequently used were mass media (54% in the 40+ years old groups). Students reported open social networks as the most used source of information (67%), while medical professionals mostly preferred official scientific channels (65%) or government/health channels (67%). Participants who are out of the labor market were more informed about COVID-19 through official channels and health organizations/authorities (57%) and open social networks (54%). We did not observe many differences between men and women regarding the preferred information sources, but we did observe a higher use of internet news portals, health authorities and scientific official channels, and close social networks in the highest educational levels compared with the lower ones. 

### 3.3. Perceptions about the Situation of the Country and the Discourse of the Authorities 

When comparing the perception of the participants about the pandemic management and situation in their own country with that of other countries in the region, most respondents considered that their countries were in a similar or better position with respect to others. The highest expression was observed in Uruguay with almost a 70% positive assessment, contrasting with 20% of Brazilian respondents (Supplementary Figure S1). What is more, about a third of the Brazilian participants considered that their country was in unfavorable conditions in terms of the pandemic management compared with the rest of the Latin American countries, and half of them answered that recommendations from health authorities at different levels were not uniform.

### 3.4. Opinions and Attitudes towards COVID-19 Vaccination 

**Figure 1:**
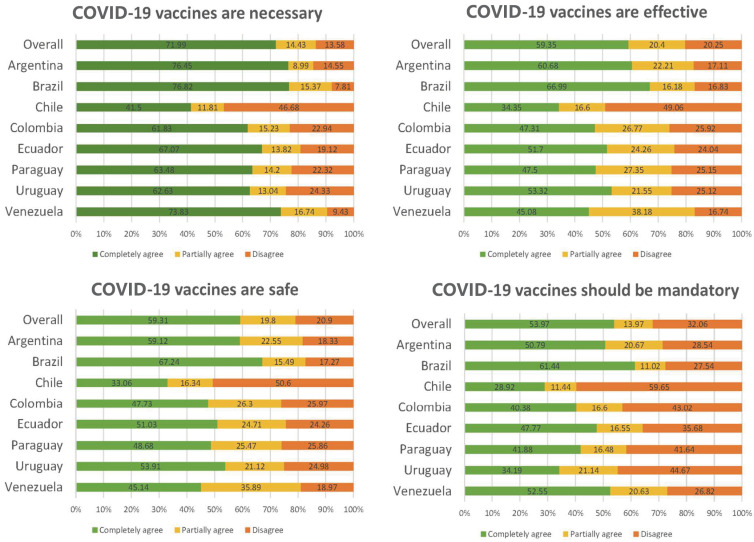
Perceptions of Necessity, Efficacy, Safety, and Mandatory vaccination of COVID-19 Vaccines.

In general, most of the surveyed people completely or partially agreed that vaccination against COVID-19 was necessary (86.4%), effective (79.8%), and safe (79.1%). Regarding compulsory vaccination, 68% of the respondents completely or partially agreed that the COVID-19 vaccine should be mandatory, while 32.1% disagreed completely (Figure 1).

These opinions were assessed on a country-specific basis, revealing, in almost all of them, results consistent with those obtained from the analysis of the total population. However, notable deviations were observed in the case of Chile, where a higher proportion of negative opinions about the vaccines was recorded, and Venezuela, with a higher percentage of positive opinions. COVID-19 vaccines encountered the highest resistance in the Chilean sample, where nearly half of the respondents did not consider them necessary. In contrast, in Brazil, Argentina, and Venezuela, most of the participants considered the vaccines as a necessity, and similar trends were observed in the other three domains (Figure 1).

### 3.5. COVID-19 Vaccination

**Figure 2:**
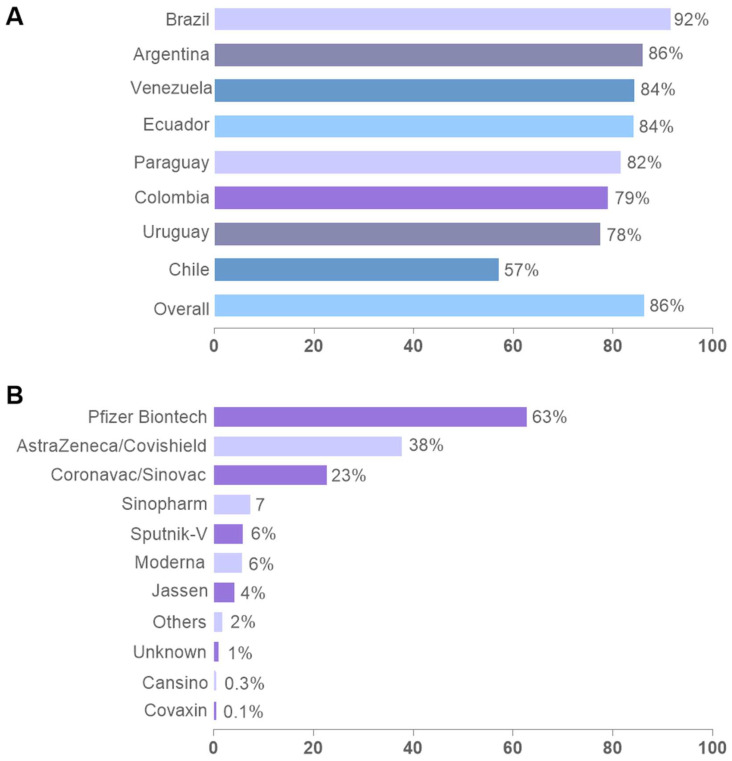
Number of vaccinated and unvaccinated individuals against COVID-19 and vaccines received by respondents in each country. Data were analyzed using the χ2-test and represented by bar graphs. (**A**) displays the respondent’s vaccination uptake by country. (**B**) illustrates the vaccines received by respondents (multiple answers were allowed).

Overall, 86% of the respondents received at least one dose of a COVID-19 vaccine. Distribution was 80.8% with a complete scheme, 2.4% with one dose and with intention to complete the primary scheme, and 3.2% with one dose without intention to complete the primary scheme. Among respondents still not vaccinated at the time of the survey (13.7%), 1.4% and 12.3% selected their intention or rejection to initiate it. The comparison of the percentage of vaccinated and unvaccinated respondents revealed statistically significant differences among countries, with Brazil being the country with the highest percentage of vaccinated participants (91.6%) and Chile being the country with the lowest percentage (57%) (Figure 2A). 

About two-thirds of all respondents indicated that they had received at least one dose of the Pfizer vaccine at the time of the survey. As observed in Figure 2B, Pfizer (63%), Astrazeneca/Covishield (38%), and Coronavac/Sinovac (23%) emerge as the three most administered vaccines. However, it is important to highlight the diversity of vaccines used throughout the region and within each country (Supplementary Figure S2). 

### 3.6. Reasons and Motivations for Vaccination

**Figure 3:**
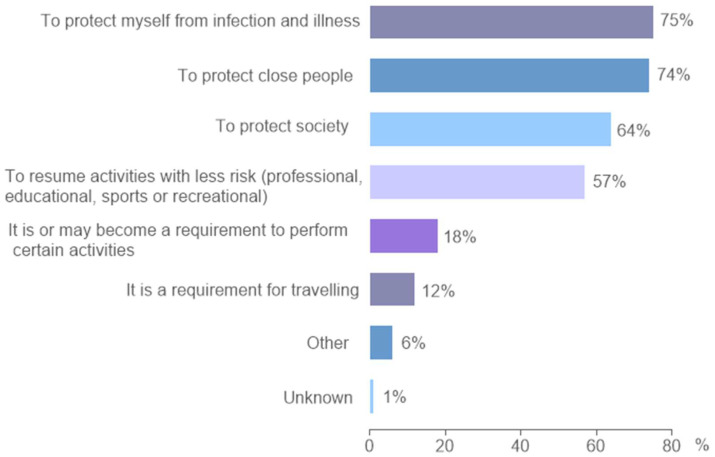
Reasons for COVID-19 vaccination acceptance. Multiple answers (up to three) were allowed.

Participants who responded that they had completed or intended to complete the vaccination were asked about the main reasons to be vaccinated (multiple choice was allowed). The most frequent reason for getting vaccinated was individual health protection (75%). However, a large proportion answered, “to protect close people” such as their relatives (74%) and society (64%), and more than a half pointed “to resume their activities with less risk” (57%). On the other hand, some of the participants accepted the vaccine because it was a mandatory requirement to carry out activities (18%) or because it could prevent them from traveling (12%) (Figure 3). Some differences were observed between countries. For example, in Brazil, a greater emphasis was placed on health and less concern on vaccination as a requirement compared with general sample, and the opposite was observed in Colombia, Ecuador and Uruguay, where the requirement to carry out activities or traveling were selected as main reasons for getting vaccinated in a higher proportion of cases than the general sample (Supplementary Figure S3). Interestingly, when studying the still-unvaccinated group but with the intention to vaccinate, it was observed that vaccinations as a requirement to engage in certain activities or for travel purposes were the main reasons for vaccination (35% and 30% of cases, respectively), while health issues were chosen in less than 30% of this group.

### 3.7. Reasons and Motivations for Not Getting Vaccinated

**Figure 4:**
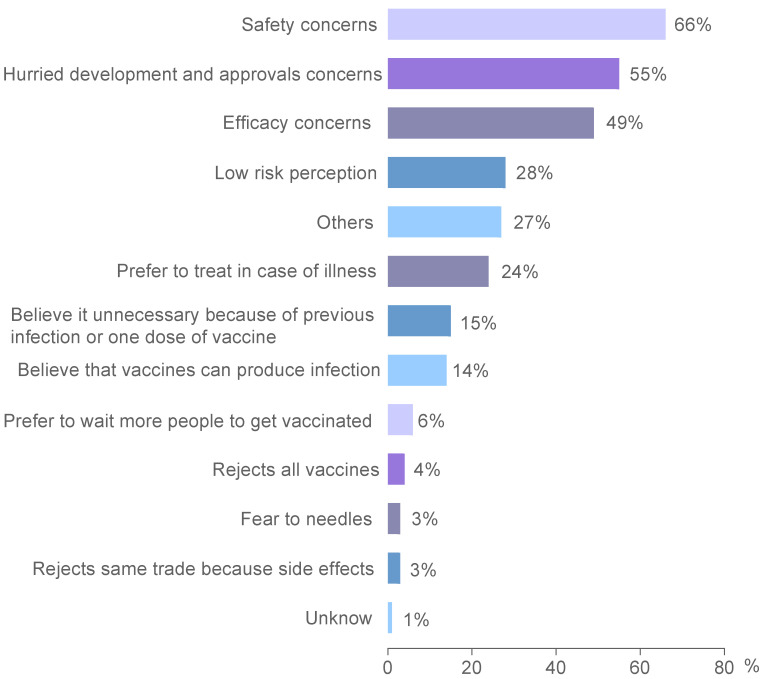
Reasons for COVID-19 vaccination rejection or hesitancy. Multiple answers (up to three) were allowed.

To identify the reasons why some individuals are hesitant or unwilling to be vaccinated against coronavirus disease, participants who answered that they had not received any vaccine against COVID-19 or were unwilling to complete the schedule were asked about the causes for not getting vaccinated. Participants’ main reasons were safety concerns (66%) and the perceived rushed development and approvals (55%), followed by concerns about effectiveness (49%) (Figure 4). There were no major differences between countries, gender, age, educational level or activity regarding this topic. 

### 3.8. Vaccine Acceptance/Rejection according to Vaccine Type

Of the 6555 participants, nearly half stated that they would take any COVID-19 vaccine, while 13% rejected all of them (reject COVID-19 vaccination in general). The highest proportion of rejections was observed in Chile and Uruguay, where 45% and 20.5% of the interviewed people rejected all vaccines. The rejection of a specific vaccine type was less prevalent, ranging from 4% for mRNA vaccines to almost 10% for the Sputnik-V vaccine, with no significant differences between countries. Slightly over 50% of the participants would be willing to be vaccinated with any of the brands approved by their countries’ health authorities.

### 3.9. Factors Related to COVID-19 Vaccination Uptake

**Figure 5:**
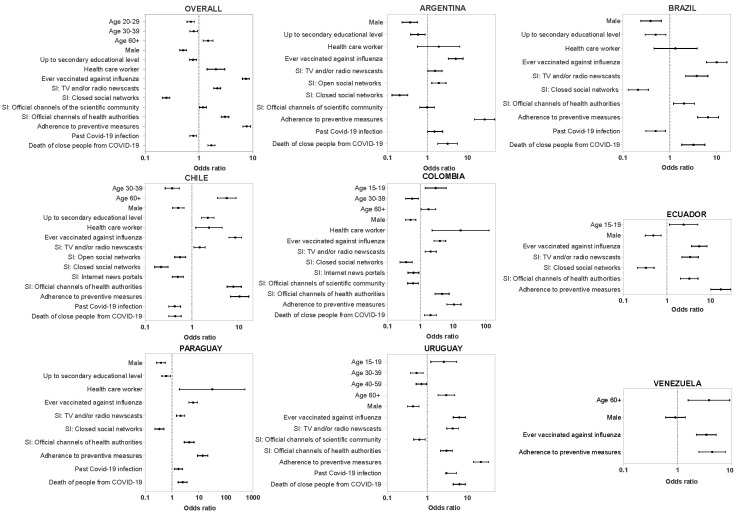
Forest plot of vaccination against COVID-19 for contextual influences. Individuals who were vaccinated compared to individuals who were not vaccinated against COVID-19. Odds-ratio (OR) and the respective 95% confidence intervals (CI) are denoted by black dots and black lines, respectively.

Statistical comparisons were performed between vaccinated and non-vaccinated groups regarding sociodemographic factors, sources of COVID-19 information, and COVID-19-related variables (Chi-square test). The results by country and overall are shown in Figure 5. Different sociodemographic factors were associated with COVID-19 vaccination uptake. Considering the 6555 participants from all countries, uptake was more frequent among the elderly (OR = 1.5; CI 95%: 1.2–1.8). Similar associations were observed in Venezuela (OR = 3.8; CI 95%: 1.6–9.3), Chile (OR = 5.54; CI 95%: 3.5–8.7), Colombia (OR = 1.78; CI 95%: 1.1–3.0), and Uruguay (OR = 3.0; CI 05%: 1.9–4.9). On the contrary, young adults between 20–29 and 30–39 years old showed a lower probability of COVID-19 vaccine uptake in the total sample (OR = 0.71; CI 95%: 0.6–0.8 and OR = 0.68; CI 95%: 0.68–0.95, respectively), Uruguay (OR = 0.54; CI 95%: 0.4–0.8, for 30–39 range), Chile (OR = 0.38; CI 95%: 0.3–0.5, for 30–39 range), and Colombia (OR = 0.56; CI 95%: 0.4–0.9). On the other hand, in Uruguay, Colombia, and Ecuador, the youngest population (15–19 years old) was more likely to get vaccinated. A lower proportion of COVID-19 vaccination uptake was observed among males in the whole sample (OR = 0.51; 95% CI: 0.44–0.59) and in each studied country. Furthermore, we observed that individuals with a lower educational level had lower odds of being vaccinated (OR = 0.8; 95% CI: 0.7–0.9), except in Chile where the opposite was shown (OR = 2.2; 95% CI: 1.6–3.0). Lastly, regarding work activity, being HCW strongly correlated with higher vaccine uptake in the whole sample (OR = 2.07 (1.42–3.01), *p* < 0.001) and in each country. The other categories of employment did not have a significant correlation. 

Vaccine uptake inversely correlated with the use of closed social networks as a source of information (OR = 0.25; 95% CI: 0.2–0.3). In contrast, the most commonly used sources of information among vaccinated people were: TV/radio news (OR = 2.2; 95% CI: 1.9–2.5), official channels of public organizations and health authorities (OR = 3.2; CI 95%: 2.6–3.6), and official channels of the scientific community (OR = 1.2; CI 95%: 1.0–1.4). 

Finally, when analyzing variables related to preventive habits and the personal or close people’s history of COVID-19 infection, it was shown that having ever received vaccination against influenza (OR = 7.46 (6.42–8.68), *p* < 0.001), using non-pharmacological preventive measures (use of masks, alcohol, and hand washing) (OR = 7.71 (6.54–9.09), *p* < 0.001), and having lost a close person to COVID-19 infection (OR = 1.71 (1.47–1.99), <0.001) were positively associated with vaccine uptake. On the contrary, participants with a history of COVID-19 showed lower odds ratios of being vaccinated against COVID-19 (OR = 0.8; CI 95%: 0.7–0.9). 

## 4. Discussion

The COVID-19 pandemic has posed significant challenges worldwide, and countries have been struggling with the complex issue of vaccine uptake. This study presents a comprehensive analysis of various factors that contribute to vaccine acceptance or hesitancy in eight countries of South America. 

Our study showed a high level of confidence and general agreement regarding the need, efficacy, and safety of vaccination against COVID-19 in the studied countries. These results are consistent with most studies in the region that investigated attitudes and perceptions towards routine vaccination prior to the pandemic and reaffirm the vaccination culture that the Region of the Americas has had for decades [21]. However, our results showed that 32% of the respondents disagreed that COVID-19 vaccination should be mandatory, and this percentage increased to 96.2% among unvaccinated individuals. Vaccination culture plays a pivotal role in shaping individuals’ decisions regarding immunization. However, in a region where mandatory vaccination for regular vaccines is the rule, objections arise at least for vaccination against COVID-19 and, in fact, in most countries it was voluntary. These findings also show the contentious nature of mandatory vaccination policies, which have sparked debates and concerns among individuals who are generally inclined toward vaccine acceptance. Those who support mandatory vaccination prioritize the concept of community disease prevention and the need for high vaccination coverage to achieve herd immunity and prevent disease even in those unvaccinated individuals [22]. On the other hand, it has been spread that mandatory vaccination violates bioethical principles (the Nurember Code), it is coercive (by compelling individuals to get vaccinated through threats of job loss or fines) and it is discriminatory (it discriminates against non-vaccinated individuals), or even that it infringes upon civil liberties (like liberty, privacy, and bodily integrity). The arguments that sustain these asseverations have no solid foundations from a moral, ethical, and logical point of view [23]. Nevertheless, policymakers should try alternative methods to encourage voluntary vaccination against COVID-19 before contemplating mandatory vaccination (i.e., information campaigns and making vaccines more easily accessible). In this sense, the World Health Organization stated that mandates should be considered only after people have been given the opportunity to get vaccinated voluntarily and/or once there is sufficient reason to believe this alone will not be enough to achieve important societal or institutional objectives [24].

In our analysis, the overall COVID-19 vaccination uptake among respondents was 86%, a proportion slightly higher than the 81.3% of people who received at least one dose of COVID-19 vaccine in South America reported by Our World in Data for the same period [20]. Compared to these reports, the population surveyed in our study reported for that moment a lower vaccine uptake in Argentina, Chile, Colombia and Uruguay and a higher vaccine uptake in Brazil, Ecuador, Paraguay, and Venezuela. This gap was less than 10% in most countries. A higher difference was observed in Chile and Paraguay instead. In Chile, vaccine uptake in our sample was 57%, contrasting with official reports that at the time of the survey there was a vaccination coverage of 91%. On the contrary, in Paraguay, vaccine uptake in our survey was 82%, while official reports for that date showed 56% coverage. It is possible that selection biases may have occurred in our study, addressing, to a greater or lesser extent, potentially anti-vaccine niches or, on the contrary, people highly committed to vaccination, which in both cases can lead to greater interest in answering this type of survey. Nevertheless, it must be considered that while overall vaccination coverage in Chile is high, certain population segments might harbor vaccine concerns or display resistance. Also, it must be noted that the 84% vaccination uptake among respondents in Ecuador could be related to the compulsory strategy that the country had adopted for COVID-19 vaccination. 

While the main reasons for getting vaccinated were individual and collective health protection, key drivers of vaccine hesitancy in our analysis were concerns about safety and the vaccine production process. This pattern was similar in all countries. Addressing these concerns is crucial for building public trust and increasing vaccine acceptance rates. Transparent communication regarding the rigorous development, testing, and regulatory approval processes is essential to assuage fears and misconceptions. Additionally, the Vaccine Safety Net of the WHO states that the goal of vaccine safety communication should be to empower people to make informed choices about COVID-19 vaccination [25]. Any communication approach must encourage trust in health authorities and those delivering the vaccine, facilitate access to timely, accurate and credible information about COVID-19 vaccination safety via trusted channels, and provide people with a means of asking questions and having their concerns addressed. Some recommendations in this regard would be to plan and prepare communication prior to vaccine introduction; set up lines of communication via trusted channels, influencers, and community leaders; identify potential threats to confidence in COVID-19 vaccine safety; listen proactively to the public; and communicate in ways that build understanding and trust, with transparency, clarity, and acknowledging uncertainty when it exists. Public health authorities must proactively and timely disclose data on adverse events, enabling individuals to make informed decisions about vaccination. Finally, communication should include messages about COVID-19 vaccine safety using an evidence-based approach. It is recommended to use pre-test messages with representatives of target audiences; work closely with the media; build a social media presence with regular communications and real-time updates; and provide detailed information on the vaccine development and approval process as well as addressing common myths and misconceptions about the vaccine. In this regard, many of these eight South American countries spread this kind of information through their official channels [26,27,28,29,30,31,32,33,34,35,36,37,38]. 

Interestingly, this study did not identify a significant preference for one vaccine over another and contrasts with the findings of Argote and colleagues, which showed a preference for Western-produced vaccines over the Chinese or Russian ones among Latin American citizens before the roll-out of mass vaccination campaigns [39]. Vaccine acceptance rates in our study were relatively consistent across available vaccine options, suggesting that once massive campaigns were implemented, concerns and hesitancy were not specific to a particular brand. Consequently, addressing overarching concerns related to vaccine safety and production processes is crucial for enhancing acceptance of all available vaccines. Comprehensive communication strategies should address common misconceptions and provide accurate information to foster confidence in the effectiveness and safety of all authorized vaccines.

Since the COVID-19 pandemic, researchers have reported influential determinants that favor vaccine refusal or hesitancy. A multinational study on the psychological causes of vaccine refusal showed that lack of trust in health authorities, fear of side effects, and low perception of personal risk have negative impacts on vaccination [40]. In this regard, 13% of our sample refused the COVID-19 vaccine, mainly because of safety and vaccine production process concerns. Lazarus and co-workers (2021) performed a survey among 23 countries worldwide and, in agreement with Hornsey et al., indicated that trust in the information provided by the governments was a positive determinant of vaccine acceptance [10]. In countries where trust in the government information was higher, subjects were more likely to take the vaccine. Although our survey did not ask specifically about this issue, we found a positive correlation between vaccine uptake and being informed through official channels. Brazil’s results are of particular interest in this regard. The Brazilian surveyed population, despite referring to a contradictory discourse of the health authorities and perceiving themselves in a worse situation than the rest of the countries in the region, had a high level of vaccine uptake, which could reflect the important vaccination culture of that country. The association found in our study between COVID-19 vaccine uptake and history of influenza vaccination may also reflect people’s vaccination culture or may just be linked to a similar population at risk. 

In our study population, it was found that individuals of older age (over 60 years) are more likely to accept vaccination in the total sample, and specifically in Chile, Colombia, Uruguay, and Venezuela. These findings are consistent with other studies conducted in Latin America. A systematic review and meta-analysis including countries in the Americas reported a high prevalence of intention to vaccinate among the elderly population (63%) [41]. The difference in age between the population that accepts or refuses vaccination is probably related to a higher perception of risk between the elderly, who were the most impacted by this disease worldwide, and a low perception in the youngest, among which the disease was usually mild and less severe. Interestingly, in Colombia, Ecuador and Uruguay, adolescents also showed a correlation with higher vaccine uptake without a clear explanation for this finding. 

Demographic characteristics have been shown to be another influential factor in vaccine acceptance. In our study, education up to the secondary level was associated with a lower COVID-19 vaccine uptake overall. We cannot assure that this global finding reflects lower risk perception, awareness, knowledge, confidence, or access. In a large-scale pre-pandemic analysis that used retrospective data drawn from 290 surveys across 149 countries, the determinants of vaccine uptake across the globe showed strong consistency with being male or having fewer years of education associated with decreased chances of uptake [42]. Similarly, in a recent survey that measured vaccination confidence and access in children caregivers in 2022 in Argentina, the educational level directly correlated with a higher vaccination access index but not with a higher vaccine confidence index [43]. Nevertheless, in our study, this correlation was not observed in all countries and, inversely, in Chile, the tertiary/university level was associated with lower COVID-19 vaccine uptake. Although we cannot prove it, it is possible that this finding is more linked to the concept of healthism, in which high-socioeconomic status individuals, i.e., those with more social, economic and educational resources available to them, are more likely to be vaccine-hesitant. This phenomenon was well described, especially in European high-income countries [44,45], and it may be the case of Chile, where it seems that a more hesitant population was somehow selected. However, in other high-income South American country such as Uruguay, this pattern was not observed. 

Regarding gender and vaccine confidence/uptake, in a systematic review that included 46 studies mainly from Europe and the US, male gender was associated with a greater likelihood of intending to accept a COVID-19 vaccine in 35 of them (58%) [46]. On the contrary, in a study that investigated Latinx communities in the US, the authors describe that men are less likely than women to receive the COVID-19 vaccine, and among the contributing barriers, they suggest that men are more vulnerable to online misinformation regarding vaccine indications, effectiveness, and safety [47]. Furthermore, another meta-analysis observed high heterogeneity in responses between countries [10]. 

In our study population, men exhibited a lower likelihood of accepting vaccination, which is in line with other local and foreign reports that show that male gender was associated with lower vaccine uptake and with the lack or delay of their children’s regular vaccination [21,42,48,49]. Some authors suggest as possible factors related to these attitudes, labor duties and other responsibilities, forgetfulness, or lower perception of risk in a population that attends medical check-ups less than women [50]. 

In the digital age, information dissemination occurs rapidly through social networks. While closed social networks are generally considered less influential than other sources of information, this study reveals their significant impact on individuals’ vaccine decision-making. Surprisingly, a substantial percentage of vaccine refusers (nearly 40%) rely on closed social networks as their primary source of information, compared to only 13% of vaccinated individuals. It is likely that the information circulating within these social media networks is unreliable, meaning that it is not certified by public health organizations but rather generated by certain anti-vaccine groups [51]. It is known that these anti-vaccine movements employ various strategies of emotive persuasion to attract readers, while also utilizing bots and trolls to rapidly propagate their messages [52]. This underscores the urgent need to address the misinformation and negative narratives circulating within these networks. Loomba and colleagues have shown how COVID-19 vaccine misinformation negatively impacted on vaccination intent, generating a decline of >6% among those who stated that they would definitely accept a vaccine to protect themselves or others in a population from the UK and USA [53]. In an inverse way, an experiment in six Latin American countries demonstrated that providing basic information about the safety and efficacy of COVID-19 vaccines, as well as priming the social approval benefits of vaccinating, increases vaccine acceptance among hesitant individuals [16]. Argote and colleagues have also shown that not only the message, but also the messenger, is of importance. In their Latin American experiment, the authors found that medical organizations’ and authorities’ endorsements remained more persuasive than those of the president or religious leaders in moving individuals to accept the COVID-19 vaccine [39]. The public’s willingness to accept a vaccine is not static; it is highly responsive to current information and sentiment around a COVID-19 vaccine, as well as the state of the epidemic and perceived risk of contracting the disease. Therefore, to combat false information in the population, it is essential that authorities take action to transmit information in a clear and concise way that is free of scientific jargon, as expressed in the Delphi Multinational consensus [54].

Thus, governments should update information regarding the determinants of their population’s COVID-19 vaccination acceptance or hesitancy to generate mass communication campaigns aimed at inducing broad public vaccine uptake to substantially reduce morbidity and mortality from COVID-19 in the region. These kinds of campaigns should be specifically targeted to more hesitant people, who resulted in being, in our study, young adults, males, and people with low educational levels. In addition, as being informed through mass media or health authorities’ channels was associated with higher vaccine uptake, these channels are suggested as the most effective for this population. 

It has been reported by other authors that attributing less importance to COVID-19 measures is a predictor of being unvaccinated, while taking the influenza vaccine is associated with being vaccinated [55,56]. Similarly, our study showed that using preventive measures and having received the influenza vaccine were positively associated with COVID-19 vaccine uptake. We believe that the higher COVID-19 vaccination acceptance among people with previously acquired prevention habits enhances the importance of routine health promotion and protection strategies, where awareness of individual disease risk and collective responsibility should be the focus of educational campaigns.

This study presents some limitations. It should be noted that most participants presented a high educational level in our sample, and a relatively low proportion of unemployed persons was observed. As the survey was carried out exclusively online and was disseminated through social media networks, these features could have biased the study, since, for example, individuals from rural areas, the very elderly, those with lower socioeconomic and/or educational levels, or others that may not have the same access to the internet were less represented in this report. On the other hand, the self-administered questionnaire methodology did not allow us to clarify questions if respondents found them confusing, and the vaccination cards of the respondents were not verified, so vaccination status was only based on self-report. However, almost all these limitations are inherent to online and self-reported surveys, which, on the other hand, have the benefit of reaching a high number of individuals in a short period of time. Our study did not assess perceptions, attitudes, or willingness to vaccinate children against SARS-CoV-2, since including this topic would have extended the questionnaire, risking the completion of the survey. Finally, we did not evaluate associations between the macro-characteristics of the countries and the results; rather, we were more interested in investigating the associations between the same population profile of any country and adherence to COVID-19 vaccination. However, this could be a topic to explore in future investigations. 

## 5. Conclusions

This article provides valuable insights into the complex dynamics surrounding vaccine acceptance in South America. Understanding these dynamics is crucial for formulating effective strategies to achieve widespread vaccine coverage and mitigate the impact of the pandemic [9]. Showing the impact of closed social networks, vaccine hesitancy patterns, vaccination culture, concerns about vaccine safety and production processes, and the importance of effective communication strategies, can help public health officials to develop targeted interventions to enhance vaccine acceptance. Our study reveals a higher COVID-19 vaccination acceptance among people with previously acquired prevention habits, highlighting the importance of routine health promotion and protection strategies. Communication campaigns through social networks stressing safety issues may help to reach hesitant people and combat misinformation.

## Figures and Tables

**Table 1:** Demographic participants’ characteristics and COVID-19-related variables.

Variable	Overall	Argentina	Brazil	Chile	Colombia	Ecuador	Paraguay	Uruguay	Venezuela
*N* (total)	6655	900	900	853	699	761	851	893	668
*N* (weighted)		749	3694	338	868	281	111	61	453
**Female gender (%)**	52.1	50.0	50.7	51.9	58.0	49.6	54.5	48.1	56.6
**Age group (%)**									
15–19	10.1	10.4	9.6	8.1	10.7	12.2	13.0	8.9	12.1
20–29	20.7	20.5	20.2	19.4	22.3	23.7	26.1	18.5	20.0
30–39	20.0	18.9	20.5	19.1	19.7	20.2	21.9	16.7	19.5
40–59	31.2	29.7	32.0	31.9	30.3	28.7	25.2	30.6	31.8
60+	18.0	20.6	17.7	21.5	16.9	15.2	13.9	25.4	16.7
**Educational level (%)**									
Primary	4.0	2.2	4.8	1.1	3.7	4.1	4.6	4.5	3.3
Secondary	30.6	33.7	28.2	30.4	35.9	33.2	28.4	48.6	30.8
Higher education	65.4	64.0	67.0	68.5	60.4	62.7	67.0	47.0	66.0
**Employment status (%)**									
Student	17.1	20.1	16.2	17.8	15.2	22.6	23.2	17.4	17.7
Housewife/retired	20.2	19.1	19.1	23.7	24.4	18.8	18.5	25.4	21.6
Unemployed	10.7	6.2	9.0	9.1	19.0	16.8	10.8	6.7	14.6
Health care worker	5.9	5.6	5.7	5.0	7.5	5.4	5.9	5.5	6.7
Employed	46.0	49.0	50.1	44.3	33.9	36.4	41.6	44.9	39.4
**COVID-19 sources of information (%)**									
TV and/or radio newscasts	51.7	52.7	51.4	52.7	52.6	52.9	51.5	56.5	49.1
Open social networks (Facebook, Instagram, Twitter, etc.)	52.8	62.8	48.2	54.8	52.9	60.3	57.8	52.3	66.7
Closed social networks (WhatsApp, Telegram)	16.1	14.7	13.7	28.5	17.7	16.8	18.1	17.8	25.3
Internet news portals	47.8	42.4	51.8	49.7	39.7	38.8	32.2	48.3	47.4
Official channels of public health authorities	43.3	50.2	43.8	36.9	40.1	44.5	45.7	48.3	37.2
Official channels of the scientific community	40.6	36.3	45.4	35.8	35.6	37.5	27.3	30.5	29.2
**Close people death from COVID-19 (%)**	43.5	31.5	48.1	30.5	38.9	43.0	41.8	15.8	48.9

## Data Availability

The data that support the findings of this study are available from the corresponding author, AU, upon reasonable request.

## Supplementary Materials

The following supporting information can be downloaded at: https://www.mdpi.com/article/10.3390/vaccines11111660/s1, Survey: Confidence in Vaccines in Latin America; Figure S1: Perception of surveyed people about the COVID-19 situation of the country compared to other countries in the Latin American region; Figure S2: Vaccines received by country; Figure S3: Main reasons to get vaccinated by country.
