# Supplementary material for: Opinions, Attitudes and Factors Related to SARS-CoV-2 Vaccine Uptake in Eight South American Countries
Source: Vaccines (Basel). 2023 Oct 30;11(11):1660. doi: 10.3390/vaccines11111660 (PMC10675814; doi:10.3390/vaccines11111660)
Supplement: Supplementary file 1 [file vaccines-11-01660-s001.zip › vaccines-2615496-supplementary.pdf]

Supplementary material

Survey.

Confidence in Vaccines in Latin America

This research is conducted by the "Confidence in Vaccines Latin America" Group, an international group of professionals working in various areas of healthcare in Latin America. Our aim is to generate ideas and strategies for public health based on the opinions and behaviours of societies. If you are 14 years old or older, your opinion is very important, and it will only take 10-15 min of your time. The information you provide will be treated confidentially and anonymously.

To learn more about the study and, its future results, please visit:

https://confianzaenlasvacunasla.org

If you agree to participate, please click the button next to 'Yes, I agree to participate' here:

Yes, I agree to participate

I do not agree to participate

1. WHICH COUNTRY ARE YOU RESPONDING FROM?

(01) Argentina

(02) Brazil

03) Chile

(04) Colombia

(05) Ecuador

(06) Paraguay

(07) Uruguay

(08) Venezuela

2. WHICH PROVINCE? (this is the example of Argentina. Answers were adapted according to each country)

(01) Buenos Aires

(02) Federal Capital

(03) Catamarca

(04) Chaco

(05) Chubut

(06) Córdoba

(07) Corrientes

(08) Entre Ríos

(09) Formosa

(10) Jujuy

(11) La Pampa

(12) La Rioja

(13) Mendoza

(14) Misiones

(15) Neuquén

(16) Río Negro

(17) Salta

(18) San Juan

(19) San Luis

(20) Santa Cruz

(21) Santa Fe

(22) Santiago del Estero

(23) Tierra del Fuego, Antarctica, and South Atlantic Islands

(24) Tucumán3. WHICH CITY? -------------------

4. GENDER

(01) Female

(02) Male

(03) Other (please specify): _______ (OPEN FIELD)

(04) Prefer not to answer

5. AGE GROUP

(01) Up to 14 years old (END OF SURVEY. This study is intended for individuals over 14 years old)

(02) 15 to 17 years old

(03) 18 to 24 years old

(04) 25 to 29 years old

(05) 30 to 39 years old

(06) 40 to 59 years old

(07) 60 years old or older

6. HIGHEST EDUCATION LEVEL ACHIEVED

(01) Primary level (completed or incomplete)

(02) Secondary level (completed or incomplete)

(03) Tertiary or university level (completed or incomplete)

7. CURRENT ACTIVITY

(01) Student (SKIP TO Q11)

(02) Homemaker or retired (SKIP TO Q11)

(03) Unemployed (SKIP TO Q11)

(04) Active Health workers

(05) Active in other professional fields (SKIP TO Q11)

8. (ONLY HEALTH WORKERS–ANSWER 04 IN Q7) How long have you been working in this field?

(01) Up to 2 years

(02) More than 2 and less than 5 years

(03) More than 5 and less than 10 years

(04) 10 years or more

9. (ONLY HEALTH PERSONNEL–ANSWER 04 IN Q7) What is your role in healthcare?

(01) Doctor

(02) Nurse

(03) Laboratory technician, diagnostic imaging, or other

(04) Community health worker

(05) Other specialties (physiotherapy/nutrition/psychosocial area)

(06) Health workers WITHOUT direct patient care (administrative, ambulance driver, stretcher bearer, teacher)

10. (ONLY HEALTH PERSONNEL–ANSWER 04 IN Q7) Is your professional link with a private or public institution?

(01) Private

(02) Public

(03) Both

11. (FOR EVERYONE) When was the last time you were vaccinated against influenza/flu?

(01) I have never been vaccinated against influenza.

(02) In 2021.

(03) In 2020.

(04) Before 2020.

12. Now let's talk about the pandemic. Have you had COVID-19?

(01) Yes, once.

(02) Yes, twice.

(03) No (SKIP TO Q14).

(04) I don't know (SKIP TO Q14).

13. (ONLY FOR THOSE WHO ANSWERED "YES" TO HAVING COVID-19–RESPONSES 01 or 02 IN Q12) Among the options I am about to mention, how severe was your case?

(01) No symptoms.

(02) Mild symptoms managed at home.

(03) Required hospitalization.

14. (FOR EVERYONE) Has any of your immediate relatives or people very close to you died from COVID 19?

(01) No.

(02) Yes, one person.

(03) Yes, more than one person.

15. Please indicate below the three (3) most used sources by you to obtain information, considering everything related to the pandemic. (ONLY MARK THREE OPTIONS)

(01) TV and/or radio news.

(02) Open social media (Facebook, Instagram, Twitter, etc.).

(03) Closed social media (WhatsApp, Telegram, etc.).

(04) Online news portals.

(05) Official channels of the scientific community (institutions, specialized journals, etc.).

(06) Official channels of public organizations and health authorities.

16. Based on the information you receive from these media sources about the pandemic, would you say that the situation in your country is better, worse, or similar to others countries in Latin American?

(01) Worse.

(02) Similar.

(03) Better.

(04) I don't know.

17. In general, would you say that public authorities at different levels (national, regional, local) provide similar recommendations to the population regarding COVID-19?

(01) Never.

(02) Rarely.

(03) Frequently.

(04) Always.

18. Throughout the pandemic, would you say that you and most of your family, friends, and known person have been adhering to the use of masks, frequent hand hygiene, and social distancing?

(01) Never.

(02) Rarely.

(03) Frequently.

(04) Always.

19. Assuming you are vaccinated or intend to get vaccinated, do you maintain/intend to maintain precautions such as wearing masks, hand washing, and using hand sanitizer?

(01) Yes.

(02) No.

(03) Maybe.

20. Please indicate whether you disagree, partially agree, agree, or strongly agree with the following statements regarding COVID-19 vaccination:

20.1. Vaccination is NECESSARY.

(01) Disagree

(02) Partially Agree

(03) Fully Agree

20.2. Vaccination should be MANDATORY.

(01) Disagree

(02) Partially Agree

(03) Fully Agree

20.3. The vaccines approved in your country are SAFE.

(01) Disagree

(02) Partially Agree

(03) Fully Agree

20.4. The vaccines approved in your country are EFFECTIVE.

(01) Disagree

(02) Partially Agree

(03) Fully Agree

21. Have you been vaccinated against COVID-19? Choose the most appropriate response based on your current situation:

(01) No, but I intend to get vaccinated.

(02) No, and I do not intend to get vaccinated (SKIP TO Q26).

(03) Yes, I have received the first dose, but I do not intend to complete the vaccination.

(04) Yes, I have received the first dose and I intend to complete the vaccination.

(05) Yes, I have received two or more doses.

(06) Yes, I have received a single-dose vaccine.

22. (ONLY FOR THOSE WHO HAVE ALREADY BEEN VACCINATED, WITH 1, 2, OR 3 DOSES–RESPONSES 03, 04, 05, 06 in Q21) Which vaccine(s) did you receive? (MULTIPLE CHOICE–THEN SKIP TO Q24)

(01) I don't know/Don't remember.

(02) Pfizer-BioNTech.

(03) Sputnik-V.

(04) Moderna.

(05) AstraZeneca/Covishield.

(06) Coronavac.

(07) Covaxin.

(08) CanSino.

(09) Janssen.

(10) Sinovac.

(99) Other.

23. On a scale of 1 to 5, with 1 being very poor and 5 being excellent, please rate your vaccination process in terms of the following aspects:

23.1. Waiting time for the appointment.

(01) Very Poor

(02) Poor

(03) Average

(04) Good

(05) Excellent

23.2. Communication of the appointment.

(01) Very Poor

(02) Poor

(03) Average

(04) Good

(05) Excellent

23.3. Waiting time at the vaccination site.

(01) Very Poor

(02) Poor

(03) Average

(04) Good

(05) Excellent

24. Have you received any vaccination certificate exclusively for the COVID-19 vaccine?

(01) Yes.

(02) No.

(03) Registration in the existing vaccination card.

25. (ONLY FOR THOSE WHO HAVE COMPLETED OR INTEND TO COMPLETE VACCINATION–RESPONSES 01, 04, 05, 06 in Q20) Which are the main reasons you have/had for getting vaccinated? (THEN SKIP TO Q27)

(01) To avoid getting infected or getting sick.

(02) To protect the people I care about.

(03) To protect society.

(04) To be able to resume my work/educational/sports/recreational activities with less risk.

(05) Because it is/could be a requirement for traveling.

(06) Because it is/could be a requirement for certain activities (work/education/sports/recreation).

(07) Other (please specify) ________________________________ (OPEN FIELD)

08) I don't know.

26. (ONLY FOR THOSE WHO HAVE NOT BEEN VACCINATED OR DO NOT INTEND TO COMPLETE VACCINATION–RESPONSES 02 or 03 in Q20) Which are the main reasons you have/had for NOT getting vaccinated? (MULTIPLE CHOICE)

(01) I am concerned about the safety of COVID-19 vaccines.

(02) I am concerned about the effectiveness of COVID-19 vaccines.

(03) I do not wish to take a second dose of the same vaccine I received.

(04) I would like to wait until more people have been vaccinated first.

(05) I believe vaccines can make me sick with COVID-19.

(06) I am confident that there will be other effective treatments soon.

(07) I am confident that I have already acquired immunity (protection) through a previous infection with the virus or through my first dose.

(08) The approval/development of the vaccine may be rushed.

(09) I have a fear of needles.

(10) I don't get any vaccines.

(11) Other (please specify) ____________________________________ (OPEN FIELD)

(12) I don't know.

27. Thinking about your decision regarding COVID-19 vaccination:

(01) You feel/felt ENCOURAGED to get vaccinated by government authorities, family, or friends.

(02) You feel/felt PRESSURED to get vaccinated by government authorities, family, or friends.

(03) You made the decision to get vaccinated independently, regardless of the opinions, pressure, or encouragement of others.

28. Is there a COVID-19 vaccine that you would not take at all, even if it is approved by health authorities?

(01) I don't know.

(02) Pfizer-BioNTech.

(03) Sputnik-V.(04) Moderna.

(05) AstraZeneca/Covishield.

(06) Coronavac.

(07) Covaxin.

(08) CanSino.

(09) Janssen.

(10) Sinovac.

(99) Other. Which one? _________________

29. Why not? ______ (OPEN FIELD)

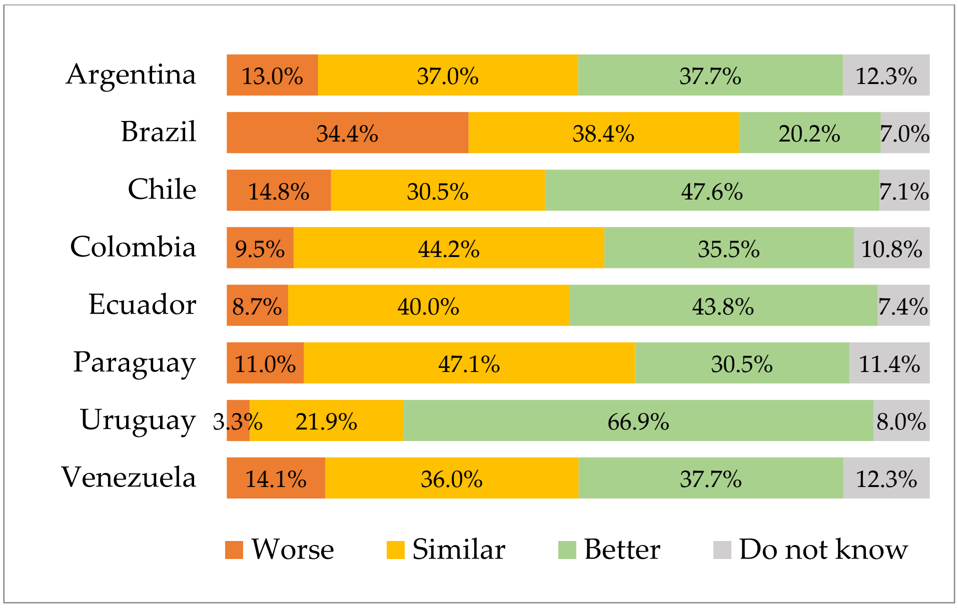

Supplementary Figure S1. Perception of surveyed people about the COVID-19 situation of the country compared to other countries in the Latin American region. (N = 6555)

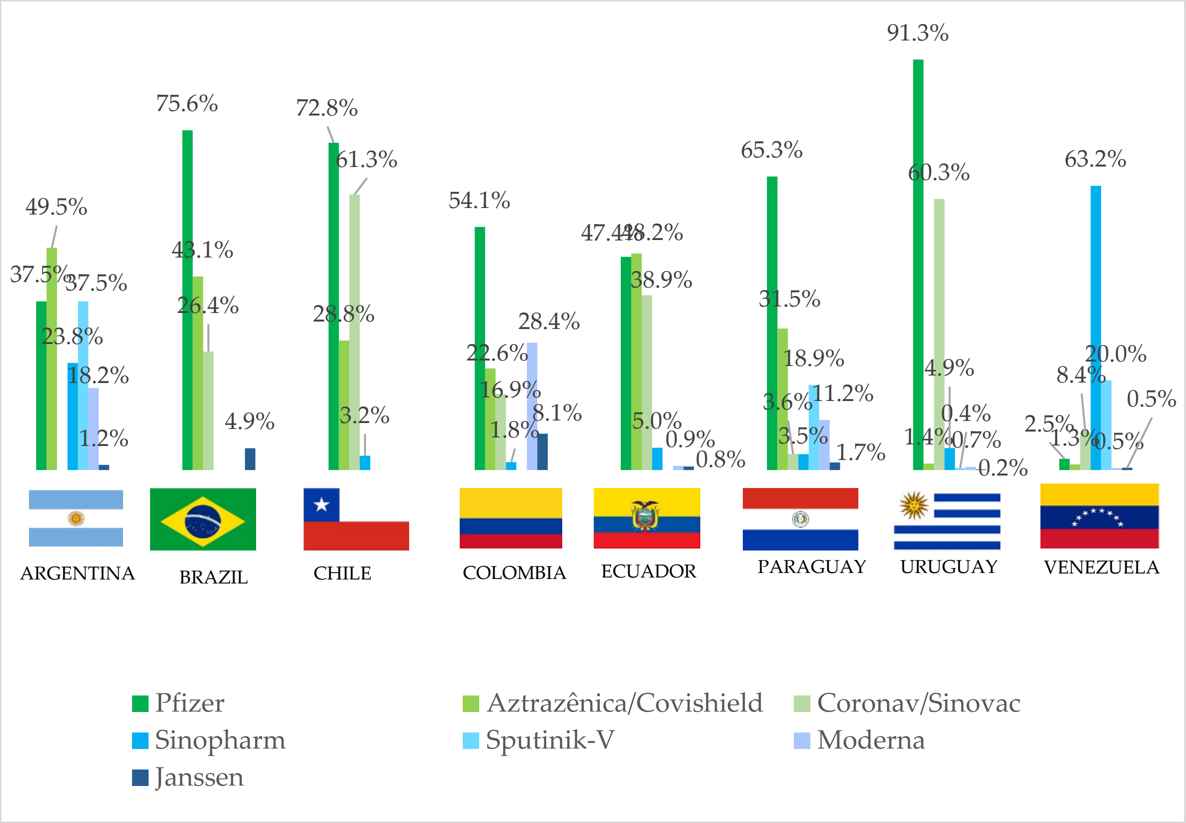

Supplementary Figure S2. Vaccines received by country. (N = 5287)

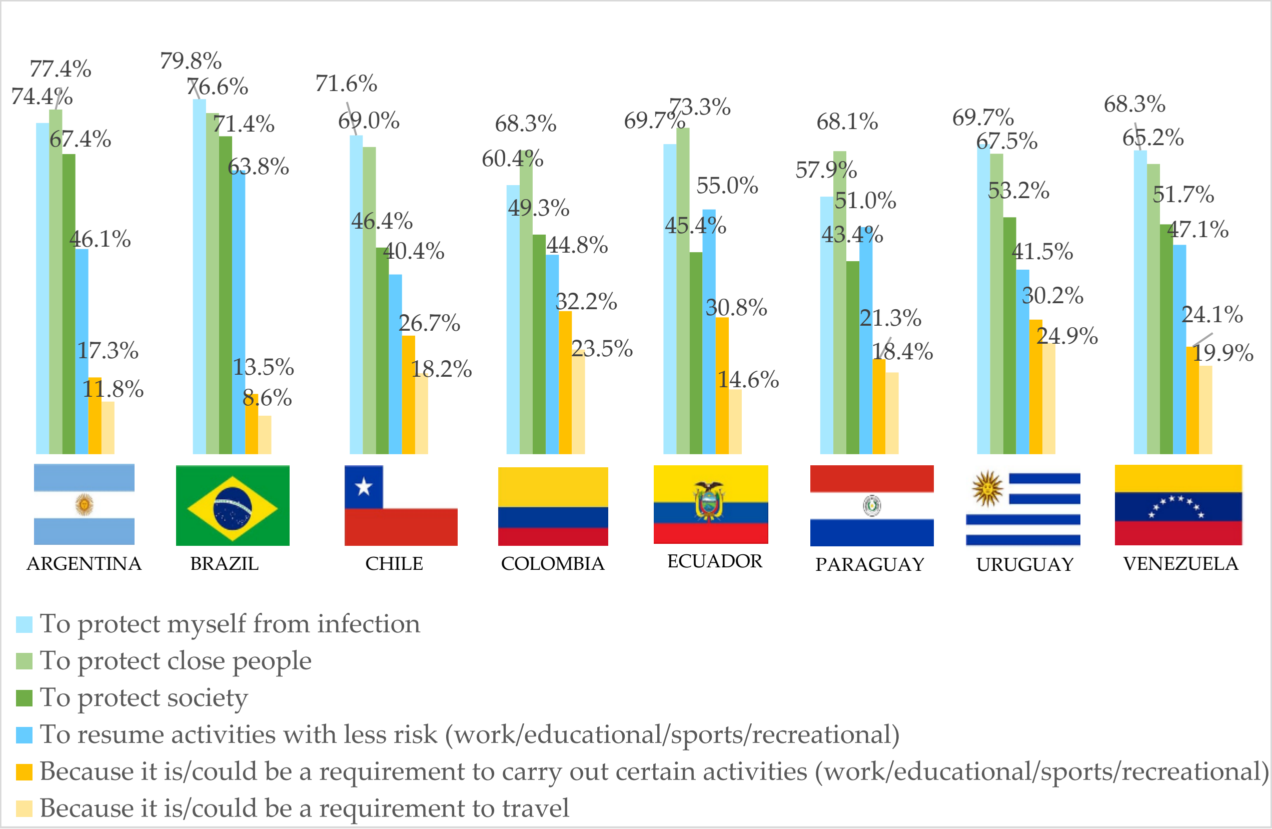

Supplementary Figure S3. Main reasons to get vaccinated by country (N = 5169)
